# Prevalence of burnout among nurses working at a psychiatric hospital in the Western Cape

**DOI:** 10.4102/curationis.v43i1.2117

**Published:** 2020-08-04

**Authors:** Anathi F. Tununu, Penelope Martin

**Affiliations:** 1 School of Nursing, Faculty of Community and Health Sciences, University of the Western Cape, Cape Town, South Africa

**Keywords:** burnout, depersonalisation, emotional exhaustion, lack of personal accomplishment, mental health care users, nurse and prevalence

## Abstract

**Background:**

Nurses are exposed to stress when working in the mental health care environment. This may be because of nurses being frontline health care providers. They develop close interpersonal relationships with mental health care users (MHCUs), which is inherent in the type of care that is provided. Mental health nursing may therefore be demanding and stressful, which could render mental health nurses susceptible to burnout.

**Objectives:**

To determine the prevalence of burnout among nurses working at a selected psychiatric hospital in the Western Cape.

**Methods:**

A quantitative, descriptive, survey design, by using simple random sampling was used to select 198 nurses employed at a psychiatric hospital in the Western Cape, South Africa. Maslach Burnout Inventory-Human Services Survey measuring emotional exhaustion, depersonalisation and personal accomplishment was used to collect the data. Domain scores were calculated, and the influence of the demographic variables on the domains was tested with independent samples Kruskal–Wallis tests and Mann–Whitney U tests.

**Results:**

The study had a 100% response rate. Most of the respondents experienced low emotional exhaustion, low depersonalisation and high personal accomplishment. Enrolled nursing assistants reported significantly higher emotional exhaustion than did the advanced psychiatric nurses and professional registered nurses. Respondents with more than 5 years of experience scored significantly higher in depersonalisation. No respondents met the criteria for burnout on all three domains.

**Conclusion:**

Maintaining a safe working environment with adequate nursing staff is recommended. Strategies to prevent burnout in the future include the provision of resources and the promotion of open communication between staff and management.

## Introduction

The mental health care working environment has been identified as a stressful environment to work in (Yoshizawa et al. 2016:3). Stressful working conditions may lead to burnout (Alenezi, McAndrew & Fallon 2019:1046; Alqahtani, Al-Otaibi & Zafar 2020:114; Ghavidel et al. 2019:3893; Kobayashi et al. 2020:2; Konstantinou et al. 2018:449; Looff et al. 2018:514). Burnout is defined as a psychological condition that leads to depletion of physical as well as mental energy; it is caused by chronic unresolved work-related stress and ineffective coping strategies (Konstantinou et al. 2018:450); and it is prevalent in occupations that work with humans under challenging situations (O’Connor, Neff & Pitman 2018:74). The most widely accepted definition of burnout is that it is a three-dimensional syndrome consisting of emotional exhaustion, depersonalisation and personal accomplishment (Maslach et al. 1986:192).

Burnout is common in many health care professions, especially nursing because nurses engage in close interpersonal relationships with people as part of their duties (Looff et al. 2018:507). The nursing profession is stressful, particularly mental health nursing, and therefore nurses working in this environment are more susceptible to burnout (Alenezi et al. 2019:1046). Mental health nurses are involved in the significant interpersonal burden of taking care of Mental Health Care Users (MHCUs) with compound emotional demands and complex and challenging mental health care conditions and their families (Ghavidel et al. 2019:3894; Konstantinou et al. 2018:450). They also fulfil the conflicting roles of caring for and exercising social control in MHCUs who were admitted to hospital involuntarily (McTiernan & McDonald 2015:209–210). Mental health nurses have intense relationships with the MHCUs and their families. They restrict MHCUs from harming themselves and others and manage difficult behaviours, although the results are less tangible than other disciplines of nursing, as care mainly takes place by means of interaction (McTiernan & McDonald 2015:210). In addition, violence by MHCUs towards other patients and nurses and MHCUs that are suicidal, present with unpredictable, unstable behaviour render mental health nursing to be demanding and challenging and may result in burnout (Ghavidel et al. 2019:3894).

Burnout has consequences that are potentially very serious for the mental health nurses, MHCUs they care for and the hospitals where the nurses are employed (Kobayashi et al. 2020:2). Burnout could affect the well-being of the mental health nurses negatively (McTiernan & McDonald 2015:208); it leads to alcohol consumption, impairment of the immunological system, musculoskeletal disorders and depressive disorders (Konstantinou et al. 2018:449), substance abuse (Ghavidel et al. 2019:3894) and suffering from irritability, tiredness, fatigue, headaches, gastrointestinal disorders, anger, exhaustion, insomnia, dyspnea and abnormal weight loss or gain (El-Azzab, Abdel-Aziz & Alam 2019:737). It could also lead to increased absenteeism (Ghavidel et al. 2019:3893; Konstantinou et al. 2018:449), job dissatisfaction (Alenezi et al. 2019:1046), increased turnover and decreased work efficiency (Alenezi et al. 2019:1045–1046; Ghavidel et al. 2019:3893–3894; Konstantinou et al. 2018:449) and increased health costs (Ghavidel et al. 2019:3894). Burnout is also associated with withdrawal from the MHCUs, which mean inability to provide therapeutic interventions, and the mental health nurses spend less time with the MHCUs and that leads to poor quality of care for the MHCUs and reduced MHCU outcomes (Alenezi et al. 2019:1046; Ghavidel et al. 2019:3894; Looff et al. 2018:507).

### Problem statement

Globally, studies (Alenezi et al. 2019:1046; Ghavidel et al. 2019:3893; Kobayashi et al. 2020:2; Konstantinou et al. 2018:449; Looff et al. 2018:506) have alluded to the escalation of work-related stress, which may lead to burnout among mental health nurses. Mental health nursing is stressful because it is a helping profession that involves close interpersonal working relationships with, as well as providing nursing care to, MHCUs with severe mental illness and complex emotional demands (Lee et al. 2015). These stressful working conditions may expose nurses working in psychiatric hospitals to burnout (Lee et al. 2015).

Burnout does not only have severe negative impact on mental health nurses but also on the MHCUs they serve and the mental health settings they work in (Kobayashi et al. 2020:2). Anecdotal evidence suggests that the nurses working in the research setting may be experiencing burnout; however, given that a study of this nature has not been conducted in this setting, it is unknown whether this is the case.

### Aim of the study

The aim of this study was to investigate the phenomenon of burnout among nurses working at the selected psychiatric hospital in the Western Cape.

### Objectives of the study

The objective of this study was to determine the prevalence of burnout among nurses working at the selected psychiatric hospital in the Western Cape.

### Definition of key concepts

**Burnout** is a psychological syndrome of emotional exhaustion, depersonalisation and lack of personal accomplishment that happen to people who work with many other people (Maslach et al. 1986:192). In this study burnout is when a respondent scores 27 or more in the emotional exhaustion domain, 13 or more in depersonalisation domain and 31 or less in personal accomplishment domain as stipulated in the scoring key of MBI-HSS (Maslach Burnout Inventory – Human Services Survey).

**Depersonalisation** is undesirable, cynical attitudes and feelings about clients; this insensitive and cruel perception of others can result workers to believe that their clients deserve their problems (Maslach et al. 1986:192). In this study depersonalisation is when a respondent scores 13 or more in the depersonalisation domain, as stipulated in the scoring key of MBI-HSS.

**Emotional exhaustion** occurs when emotional resources are worn out, whereby the workers feel they are no longer able to give of themselves at a psychological level (Maslach et al. 1986:192). In this study emotional exhaustion is highlighted when a respondent scores 27 or more in the emotional exhaustion domain as stipulated in the scoring key of MBI-HSS.

**Lack of personal accomplishment** is the tendency to see yourself negatively, regarding your work with the clients; workers may feel unhappy about themselves and dissatisfied with their accomplishments in the job (Maslach et al. 1986:192). In this study lack of personal accomplishment is highlighted when a respondent scores 31 or less in the personal accomplishment domain as stipulated in the scoring key of MBI-HSS.

A **MHCU** is a person who receives care, treatment and rehabilitation services or utilising a health service at a health establishment meant to improve the mental health status of a user (Republic of South Africa [RSA] 2002:6). In this study an MHCU is a patient that is admitted or was admitted for care, treatment and rehabilitation to the selected psychiatric hospital.

**Nurse** is defined as an individual that is registered to practise nursing or midwifery (Council, S.A.N. 2005:6). In this study a nurse is a person who is registered or enrolled to practise nursing at the selected hospital.

## Research design and method

### Research design

A quantitative, descriptive survey design was conducted.

### Setting

This study was conducted in one of four psychiatric hospitals in the Western Cape province, South Africa. The selected psychiatric hospital is the largest in the Western Cape and is situated in the Cape Town suburb of Mitchells Plain. The hospital has a 722-bed capacity. There are 409 nurses working at the hospital.

### Population and sampling

All the nurses working at the selected psychiatric hospital formed the target population, as they were responsible for providing direct nursing care to the MHCUs. The sample size for a survey was calculated by using the following parameters: confidence level = 95%, confidence interval = 5%, percentage 50% and population size = 409, yielding an estimated sample size of 198 (Creative Research Systems 2012:1). The researcher distributed 198 questionnaires, and all the questionnaires were answered (100% response rate), of which 167 questionnaires were valid and 31 were invalid. Simple random sampling was used to select the respondents by using the duty register as the sampling frame. Respondents were randomly selected from each nursing category. A total of six respondents per ward were selected comprising two per category and two others.

### Inclusion criteria

The inclusion criteria were all permanent nursing staff providing direct care.

### Exclusion criteria

The exclusion criteria were the various nursing managers (director, area, operational), occupational health and safety professional nurse and the clinical coordinator, as they were not involved in direct patient care. Community service practitioners and agency nurses were also excluded as they were contract workers and, therefore, had varied tenure at the selected hospital.

### Data collection instrument

A 28-item structured self-administered questionnaire was used to collect data. The questionnaire required about 10–15 min to complete. The questionnaire included six demographic variables, namely age, race, gender, nursing rank, years of experience and functional business unit and the MBI-HSS. The scale has 22 items and uses a 7-point Likert scale (rating from ‘never’ to ‘every day’) to measure three domains of burnout, namely emotional exhaustion (9 items), depersonalisation (5 items) and lack of personal accomplishment (8 items) (Maslach et al. 1986:193).

### Validity and reliability

In this study, Cronbach’s alpha was used to determine the reliability of the instrument. The Cronbach’s alpha score for this study was 0.794. Various studies reported Cronbach’s alpha scores that are between satisfactory to high when using MBI-HSS. Alqahtani et al. (2020:111) reported a Cronbach score of 0.80 for the MBI-HSS with a range of 0.79–0.81 for the subscales. Looff et al. (2018:508) had the following alpha coefficients for emotional exhaustion, depersonalisation and personal accomplishment, 0.64, 0.81 and 0.86, respectively. In a study conducted by El-Azzab et al. (2019:733), the Cronbach alpha was 0.89. Cronbach score for emotional exhaustion, depersonalisation and personal accomplishment of a study conducted by McTiernan and McDonald (2015:211) were 0.92, 0.83 and 0.72, respectively. The questionnaire was not originally developed in South Africa, and to assess the reliability in this context a pretest was conducted and internal consistencies were calculated. The Cronbach’s alphas for this study are 0.90 for emotional exhaustion, 0.79 for depersonalisation and 0.71 for lack of personal accomplishment, all showing acceptable internal consistency (> 0.70).

### Data collection process

Data were collected from May until June 2017 for a period of 4 weeks. The researcher visited the different wards at the selected psychiatric hospital and requested permission from the sister in charge of each ward, for access to collect data. Once permission was granted, the researcher addressed all the nursing staff to establish a suitable time to explain the study and distribute questionnaires. After explaining the study and obtaining volunteers to participate in the study, the researcher left the questionnaires, information sheets and consent forms with the participants who had agreed to complete them in their own time. This was to allow them enough time to read the information sheet, sign the consent forms and complete the questionnaires. Each respondent was provided with an envelope to insert the completed questionnaires and signed consent forms, to ensure anonymity. These were then cast into a box. The researcher collected the boxes from the various wards on the day and at the time agreed upon by the respondents.

### Data analysis

The data were captured into SPSS, version 24. Age and years of experiences were recoded into categorical variables (20–29, 30–39, 40–49 and 50 and over years; and 5 years and less and more than 5 years of experience). Total domain scores were calculated and recoded by using the Maslach et al. (1986) scoring key (Table 1). For the emotional exhaustion and depersonalisation domains, high mean scores reflect high levels of burnout, and low mean scores reflect low levels of burnout, whereas for personal accomplishment domain, high mean scores reflect low levels of burnout, and low mean scores reflect high levels of burnout (Maslach et al. 1986:193). A respondent can be diagnosed with burnout syndrome, if the score is 27 or more in the emotional exhaustion domain, 13 or more in the depersonalisation domain and 31 or less in the personal accomplishment domain (see Table 1 for scoring key). Frequencies were used to the proportion of respondents scoring low, moderate and high on emotional exhaustion, depersonalisation and personal accomplishment, as well as the mean scores of emotional exhaustion, depersonalisation and lack of personal accomplishment. Independent sample Kruskal–Wallis (K) and Mann–Whitney U tests (U) were used to determine the differences in domain scores and demographic variables.

**TABLE 1:** Burnout scoring key.

Levels of burnout	Emotional exhaustion domain	Depersonalisation domain	Personal accomplishment domain
Low	0–16	0–6	0–31
Moderate	17–26	7–12	32–38
High	27 and over	13 and over	39 and over

*Source:* Maslach, C., Jackson, S.E., Leiter, M.P., Schaufeli, W.B. & Schwab, R.L., 1986, *Maslach burnout inventory*, vol. 21, p. 194, Consulting Psychologists Press, Palo Alto, CA.

### Ethical considerations

Ethical approval to conduct this study was obtained from the Bio Medical Research Ethics Committee of University of the Western Cape (UWC), and permission to conduct this study in the selected psychiatric hospital was obtained from the Research Committee of the Western Cape Department of Health. Permission to access the nurses for data collection was obtained from the Chief Executive Officer of the selected psychiatric hospital. Prior to data collection, respondents were informed that they had a right to withdraw from the study at any time, without providing reasons. Participation in the research study was entirely voluntary. All ethical principles were adhered to, and contact details of the free service for government employees namely the Independent Counselling and Advisory Service (ICAS) were given to all the respondents should they have required counselling from participating in the study. Counselling with the counsellor was prearranged by the researcher (Ethical Clearance Number: BM/17/1/4, 16 January 2017).

## Results

### Demographics

The selected samples all responded to the survey, but the completion of items on the questionnaire was inconsistent. More than half of the respondents were female (120, 61.5%) and 75 (38.5%) were male. The average age of the respondents was 41.7 years (standard deviation [SD] 10.4), ranging from 22 to 63 years with 67 (33.8%) aged 30–39 years, followed by 63 (31.8%) 50 and older, 43 (21.7%) 40–49 years and lastly 25 (12.6%) 20–29 years old. The highest nursing category of respondents were enrolled nursing assistants (81, 41.3%), followed by advanced psychiatric nurses (42, 21.4%), general professional nurses (38, 19.4%) and 35 (17.9%) enrolled nurses. The respondents had an average of 7 years (SD 10.4) of experience (median of 5 years, range < 1 to 37 years) with 107 (54.0%) with 5 years or less and 89 (44.9%) with more than 5 years’ experience. Nearly half of the respondents were from the general adult psychiatry (78, 39.4%) wards, followed by 76 (38.4%) from intellectual disability psychiatry, 35 (17.7%) from forensic psychiatry and 9 (4.5%) from child and adolescent psychiatry wards.

### Emotional exhaustion

The average score for emotional exhaustion was 15.5 (SD 11.4) (95% confidence interval [CI] 13.3–16.7) (Table 2), with more than half of the respondents in the low category for emotional exhaustion; 112 (61.9%) and 30 (15.2%) met the criteria for burnout on this domain (Table 3). Item analysis of the statement ‘*working with people all day long requires a great deal of effort’* was rated the highest (3.4 [95% CI 3.1–3.7]) and statements ‘*It stresses me too much to work in direct contact with people’* (0.9 [CI 0.7–1.1]) and *I feel like I am at the end of my rope* (0.8 [CI 0.6–1]) were rated the lowest.

**TABLE 2:** Burnout domains and individual items.

Domain and items	Mean	SD	95% CI
**Emotional exhaustion (/9)**	**15.5**	**11.4**	**13.3–16.7**
Working with people all day long requires a great deal of effort.	3.4	2.4	3.1–3.7*
I feel emotionally drained by my work.	2.1	1.9	1.8–2.4
I feel I work too hard at my job.	2.1	2.1	1.8–2.4
I feel tired when I get up in the morning and have to face another day at work.	1.9	1.9	1.7–2.2
I feel frustrated by my work.	1.6	1.7	1.3–1.8
I am at the end of my patience at the end of my work day.	1.3	1.7	1–1.5
I feel like my work is breaking me down.	1.3	1.7	1.1–1.6
It stresses me too much to work in direct contact with people.	0.9	1.4	0.7–1.1*
I feel like I’m at the end of my rope.	0.8	1.6	0.6–1*
**Depersonalisation (/5)**	**4.0**	**4.5**	**3.4–4.7**
I have the impression that my patients/clients make me responsible for some of their problems.	2.2	2.3	1.9–2.5*
I feel I look after certain patients/clients impersonally, as if they are objects.	0.7	1.5	0.5–0.9
I have become more insensitive to people since I’ve been working.	0.7	1.4	0.5–0.9
I’m afraid that this job is making me uncaring.	0.3	1	0.2–0.5
I really don’t care about what happens to some of my patients/clients.	0.1	0.5	0.1–0.2
**Personal accomplishment score (/8)**	**40.1**	**8.5**	**38.9–41.3**
I look after my patients’/clients’ problems very effectively.	5.5	1.2	5.3–5.7
I am easily able to create a relaxed atmosphere with my patients/clients.	5.3	1.3	5.1–5.5
Through my work, I feel that I have a positive influence on people.	5.3	1.5	5–5.5
I am easily able to understand what my patients/clients feel.	5.2	1.4	5–5.4
In my work, I handle emotional problems very calmly.	5.1	1.6	4.9–5.3
I feel refreshed when I have been close to my patients/clients at work.	4.9	1.6	4.6–5.1
I feel full of energy.	4.6	1.6	4.4–4.8
I accomplish many worthwhile things in this job.	4.1	2.2	3.8–4.4

*Source:* The questions used are taken from Maslach, C., Jackson, S.E., Leiter, M.P., Schaufeli, W.B. & Schwab, R.L., 1986, *Maslach burnout inventory*, vol. 21, pp. 3463–3464, Consulting Psychologists Press, Palo Alto, CA. The data is from the authors study.

SD, standard deviation; CI, confidence interval.

There were no significant differences in emotional exhaustion by gender (*U* = 0.419, *p* = 0.675), race (*K* = 2.9, *p* = 0.229), years of experience (*U* = 0.528, *p* = 0.597), age group (*K* = 5.1, *p* = 0.161) and ward (*K* = 4.1, *p* = 0.248). Enrolled nursing assistants scored significantly lower on emotional exhaustion than advanced psychiatric nurses and professional registered nurses (11.7 [SD 9.3] vs. 17, 6 [SD 11.3] vs. 20.19 [SD 13.8]) (*K* = 13.8, *p* = 0.004).

### Depersonalisation

The average score for depersonalisation was 4.0 (SD 4.5) [95% CI 3.4–4.7] (Table 2) with more than three quarters of the respondents in the low category for depersonalisation 161 [81.3%] with 9 [4.5%] of the respondents meeting the criteria for burnout on this domain (Table 3). The statement ‘*I have the impression that my patients/clients make me responsible for some of their problems’* (2.2 [CI 1.9–2.5]) was rated higher by respondents.

**TABLE 3:** Burnout domains by categories.

Scores	Emotional exhaustion (*n* = 181)		Depersonalisation (*n* = 194)		Personal accomplishment (*n* = 192)	
	*N*	%	*N*	%	*N*	%
Low	112	56.6	161	81.3	23	11.6
Moderate	39	19.7	24	12.1	37	18.7
High	30	15.2	9	4.5	132	66.7

*Source:* The burnout scoring key was taken from Maslach, C., Jackson, S.E., Leiter, M.P., Schaufeli, W.B. & Schwab, R.L., 1986, *Maslach burnout inventory*, vol. 21, pp. 3463–3464, Consulting Psychologists Press, Palo Alto, CA. The data is from the first author’s masters theses findings.

### Personal accomplishment

The average score for personal accomplishment was 40.1 (SD 8.5) [95% CI 38.9–41.3] (Table 2) with nearly three quarters of the respondents scoring high on personal accomplishment 132, [68.8%] and 23 [11.6%] meeting the criteria for burnout on this domain (Table 3). There were no significant differences in depersonalisation by gender (*U* = 1.2, *p* = 0.241), nursing category (*K* = 5.6, *p* = 0.133), years of experience (*U* = 0.26, *p* = 0.792), age group (*K* = 0.5, *p* = 0.961) and ward (*K* = 4.9, *p* = 0.182).

Overall, no respondents met the criteria for burnout on all three domains.

There were no significant differences in depersonalisation by gender (*U* = 1.9, *p* = 0.053), nursing category (*K* = 1.8, *p* = 0.617), years of experience (*U* = 0.53, *p* = 597), age group (*K* = 6.8, *p* = 0.076) and ward (*K* = 1.3, *p* = 0.710). There were differences in the depersonalisation by years of experience with staff with more than 5 years’ experience scoring significantly lower than staff with 5 years or less experience (3.15 [SD 3.7] vs. 4.76 [SD 4.9], *U* = 2.4, *p* = 0.015).

## Discussion

The results of this study revealed that the nurses at the selected psychiatric hospital did not suffer from emotional exhaustion. A study conducted with Saudi Arabian mental health nurses revealed high levels of emotional exhaustion (Alenezi et al. 2019:1050). In Greece, a study conducted among mental health nurses revealed high emotional exhaustion (Konstantinou et al. 2018:452). In Dublin, Ireland, a study conducted among hospital and community mental health nurses revealed moderate emotional exhaustion (McTiernan & McDonald 2015:213). This study results differ from the studies mentioned.

McTiernan and McDonald (2015:213) reported low levels of depersonalisation amongst nurses. These results are similar to the results of the current study. In a study performed by Alenezi et al. (2019:1050) in Saudi Arabian mental health nurses, it was reported that they had high levels of depersonalisation. In a study conducted with Greece mental health nurses, it was observed that most of the respondents reported moderate levels of depersonalisation (Konstantinou et al. 2018:452). The results of these studies are dissimilar to the results of this study.

In a study conducted with mental health nurses in Saudi Arabia, the results revealed low levels of personal accomplishment (Alenezi et al. 2019:1050). A study conducted with Greece mental health nurses revealed low levels of personal accomplishment (Konstantinou et al. 2018:452). In Dublin, Ireland, a study conducted with hospital and community mental health nurses observed moderate personal accomplishment scores (McTiernan & McDonald 2015:213). The results of these studies differ from the results of this study.

A study conducted in a Saudi Arabian mental health facility showed high levels of emotional exhaustion, high levels of depersonalisation and low levels of personal accomplishment; therefore, overall burnout score for this sample was high (Alenezi et al. 2019:1046). Konstantinou et al. (2018:452) conducted a study in Greece mental nurses and found emotional exhaustion that was high, depersonalisation that was moderate and personal accomplishment that was low; therefore, the overall burnout score for these nurses was moderate to high. In Dublin, Ireland, a study on burnout conducted with hospital and community mental health nurses revealed that both groups scored moderate for emotional exhaustion and personal accomplishment and low for depersonalisation (McTiernan & McDonald 2015:213). Therefore, none of these study show low overall burnout levels and that indicates that they differ with the results of the current study.

There are many differences between the existing literature and the finding of the current study, and this might be because of different factors such as demographic and environmental factors; a similar study was never conducted in South Africa.

The mental health nurses of the selected psychiatric hospital might be experiencing low levels of burnout because of age, as the average age for the sample of this study was 41.7 years, which is high. El-Azzab et al. (2019:734) reported a negative correlation between burnout and age, meaning that as the age increases the burnout levels are more likely to decrease.

The sample of this study might not be experiencing high levels of burnout because of the high number of females (61.5%) compared with males (38.5%). Konstantinou et al. (2018:453) state that male nurses experience more emotional exhaustion and depersonalisation, compared with female nurses. Male gender is a predictor of burnout (Kobayashi et al. 2020:2).

The nurses of this study may be experiencing low levels of burnout because of the nursing rank. Advanced professional nurses made up 21.4% of the respondents, general professional nurses made up 19.4% of the respondents, enrolled nurses made up 17.7% of the respondents and enrolled nursing assistants made up 41.3% of the respondents. This shows that the majority of the respondents were enrolled nursing assistants, which is the lowest rank compared with other ranks, and according to Kobayashi et al. (2020:2) professional seniority contributes to high levels of burnout.

The average years of experience for the mental health nurses of the selected psychiatric hospital were 9.9 years. This is a high number for years of experience, which could be another reason for the low levels of burnout in this study’s sample. Alqahtani et al. (2020:111) state that more experienced mental health nurses experience less burnout than less experienced mental health nurses.

The results of the current study showed a mean of 15.05 for emotional exhaustion, and according to Scoring & Interpretation Key-MBI-HSS (1981:1), a mean score between 0 and 16 indicates low levels of emotional exhaustion, a mean score of 4.03 for depersonalisation, and according to Scoring & Interpretation Key-MBI-HSS (1981:1), a mean score between 0 and 6 indicates low levels of depersonalisation and a mean score for personal accomplishment was 40.11, and according to Scoring & Interpretation Key-MBI-HSS (1981:1), a mean score of 39 and more indicates high personal accomplishment. The mental health nurses of the selected psychiatric hospital do not experience burnout because they scored low on emotional exhaustion, low on depersonalisation and high for personal accomplishment.

### Limitations

This study was conducted at one of the four psychiatric hospitals in the Western Cape; therefore, the results cannot be generalised for all nurses working in psychiatric hospitals in the Western Cape.

There is a paucity of literature regarding the prevalence of burnout among mental health nurses, with recent research studies only being conducted in Asia and Europe.

### Recommendations

#### Clinical practice

Burnout can be prevented by being cognisant of the following issues: maintain a safe working environment to prevent incidents; employ adequate nursing staff to prevent work overload; provide resources to prevent stress caused by the shortage, or unavailability thereof; promote open communication to allow team work; rotate nursing staff to work in other wards for professional development, avoidance of boredom, as well as exposure to different environments and challenges, which will keep them interested to learn and perform productively; and managers should support, supervise, appreciate and value nursing staff to prevent burnout.

#### Nursing education

The nursing staff should attend in-service training programmes that deal with stress management to acquire the necessary skills to cope with work stress. In addition, the nursing staff should attend courses and in-service training that update their knowledge to be competent health service providers.

#### Research

This study should be replicated in other settings, to obtain an overview of the burnout profile of health care workers, working in health care institutions in South Africa.

## Conclusion

The aim of this study was to investigate burnout among nurses working at the selected psychiatric hospital in Western Cape. According to the findings of this study, nurses working at the selected psychiatric hospital do not suffer from burnout.
